# The safety of meperidine prescribing in older adults: A longitudinal population-based study

**DOI:** 10.1186/s12877-016-0275-5

**Published:** 2016-05-11

**Authors:** Kevin J. Friesen, Jamie Falk, Shawn Bugden

**Affiliations:** College of Pharmacy, Faculty of Health Sciences, University of Manitoba, 750 McDermot Avenue, Winnipeg, MB R3E 0T5 Canada

**Keywords:** Meperidine, Pethidine, Safety, Age, Geriatrics, Utilization

## Abstract

**Background:**

Meperidine (pethidine) is an opioid analgesic that offers little advantage relative to other opioids and several disadvantages including limited potency, short duration of action, and the production of a neurotoxic metabolite (normeperidine) with a long half-life. Older adults are more sensitive to meperidine’s side effects and may have diminished renal function which leads to the accumulation of normeperidine. The Institute for Safe Medication Practices has suggested avoiding meperidine in older adults, limiting its dose (≤600 mg/day) and duration of use (≤48 h). The objective of this study was to determine the level of meperidine use in older adults and assess the dosage and duration of meperidine with reference to these safety recommendations.

**Methods:**

A longitudinal study using administrative healthcare data was conducted to examine meperidine utilization and levels of high dose and extended duration prescribing among persons ≥65 years of age between April 1, 2001, and March 31, 2014 in Manitoba*,* Canada*.* The number of meperidine prescriptions, users, duration of treatment, defined daily doses (DDD) dispensed and number of prescribers were determined over the study period.

**Results:**

In the Manitoba older adult population there was a marked decline in meperidine users and prescriptions from 2001 to 2014. There was an average use of 26.4 (95 % CI 24.0–28.8) DDDs of meperidine per user per year. While only 3.7 % of the prescriptions exceeded the 600 mg maximum daily dose, 96.7 % of prescriptions exceeded the recommended 2 days of therapy. For the remaining users of meperidine, the amount of meperidine used per person rose from 18.98 to 56.14 DDDs/user/year over the study period. The number of prescribers of meperidine declined throughout the study, but low DDD prescribers declined more quickly than high DDD prescribers.

**Conclusions:**

While meperidine use has declined, the remaining use appears to be decreasing in safety, with more meperidine prescribed per user. This seems to be driven by the continued prescribing by a small number of high DDD prescribers. Targeted educational initiatives directed at this small group of prescribers may be helpful. Alternatively removing meperidine from medication insurance schemes may provide additional incentive to avoid meperidine in older adults.

## Background

Meperidine (pethidine) was once one of the most commonly used opioid analgesics in North America [1]. More than 50 years after its introduction the American Pain Society began pointing out the problems with meperidine and suggesting limiting its use [2]. Meperidine has a rapid onset of action which makes it more prone to abuse than other opioids [3–5]. In terms of efficacy, meperidine lacks potency and has a relatively short duration of action (2.5 to 3.5 h) compared to morphine [6]. As such, it is frequently under-dosed and given at intervals longer than its duration of action resulting in poor pain control [7]. While meperidine has a half-life of only 2 to5 h, its metabolism produces normeperidine, a neurotoxic metabolite with a much longer half-life of 15 to 30 h [6]. This creates a clinical dilemma where the dosing frequency necessary for adequate pain control is likely to produce accumulation of normeperidine which has been associated with delirium and seizures.

There are a number of reasons why older adults may experience more adverse effects related to meperidine. Firstly, older adults are more sensitive to the central nervous system side effects of meperidine including anxiety, hallucinations, confusion, and seizures [6, 8]. Secondly, renal function generally diminishes with age, and as normeperidine is eliminated renally, older adults are more likely to accumulate normeperidine with repeated dosing, putting them at elevated risk of toxicity. It is therefore not surprising that meperidine has been included on the Beers list of medications that are best avoided in older adults [9, 10]. A recent examination of the outcomes associated with medications on the Beers list prescribed to older adults found meperidine to be the medication most strongly associated with unplanned hospitalizations (OR 2.37, 95 % CI 1.25–4.50) [11].

There has been further action to limit the use of meperidine. In Canada, the Institute for Safe Medication Practices (ISMP) issued safety warnings in 2004 and 2005, recommending that meperidine be limited to situations where alternative opioids are contraindicated [12, 13]. When its use cannot be avoided the ISMP recommendations suggest that doses should not exceed 600 mg of meperidine in 24 h, and duration of use should be limited to 48 h.

Given the higher risk in older adults we examined the use of meperidine in people 65 years of age and older in a longitudinal utilization analysis in Manitoba, Canada. The objective of this study was to determine the level of meperidine use in older adults and assess the dosage and duration of meperidine with reference to these safety recommendations.

## Methods

A longitudinal study using administrative healthcare data was conducted to examine meperidine utilization among persons 65 years of age and older between April 1, 2001 and March 31, 2014 in the province of Manitoba*,* Canada*.* Prescription data were obtained from the Manitoba Drug Program Information Network (DPIN) database through the Manitoba Centre for Health Policy (MCHP) at the University of Manitoba. DPIN is a centralized system used to process all outpatient prescriptions in Manitoba. This linked data provides relatively complete population data for Manitoba’s 1.3 million people and is routinely used in administrative database studies [14–16].

The number of oral meperidine prescriptions, users, days of treatment as prescribed, and defined daily doses (DDD) dispensed were determined. In Canada oral meperidine is only available as a 50 mg tablet. The World Health Organization DDD represents the assumed average maintenance dose for a drug used for its main indication in adults. The DDD value for meperidine is 400 mg which is equivalent to 50 mg given every 3 h [17].

Results were summarized across the overall study period, and were examined within each fiscal year (April 1^st^ to March 31^st^). Prescriptions and users rates were presented per 1000 persons based on Manitoba Health Registry data. Results were also stratified into age groups (65–74, 75–84, and ≥85 years) based on the age at the time of dispensation. The daily dose was calculated using prescription quantity multiplied by strength and divided by the duration of prescription. All pharmacists are required to enter the estimated days supply based on the prescription’s quantity and directions. The duration of prescription was taken as the lesser of the days supply field as entered by the dispensing pharmacist, or the number of days to when the next prescription was filled. Prescriptions were classified as high dose if they exceeded 600 mg per day. Users were classified as high dose users overall if they had one or more high dose prescriptions.

Prescriptions were considered to exceed the duration safety recommendation if the duration was more than 2 days. However, since the days supply field may not be accurately entered by the dispensing pharmacists, the prescription duration was also assessed by converting prescription quantities into DDDs. Prescriptions that exceeded 2 DDDs were considered to have exceeded the safety recommendation to limit use to 2 days of therapy.

The prescribers of meperidine were also studied. A cumulative frequency distribution of meperidine prescribers was created to determine the number of prescribers responsible for 50 % of meperidine prescribing. The proportion of prescribers prescribing larger quantities of meperidine was also examined on an annual basis. Where greater than 50 % of an individual prescriber’s prescriptions were for more than 6 DDDs, prescribers were classified as high DDD prescribers. Six DDDs represents a prescription quantity of 2400 mg or twice the ISMP recommended maximum (600 mg/day for 2 days). Prescribers who fell below this threshold were classified as low DDD prescribers.

Data analysis was done using SAS 9.4® (SAS Institute Inc., Cary, NC, USA). Univariate and least squares regression analysis were used to describe and analyze our data. For age-stratified data, ANOVA was used to compare annual rates between age groups with the Tukey method used to make pairwise comparison where the ANOVA showed a significant overall difference between the groups.

Approval for this study was obtained from the University of Manitoba’s Health Research Ethics Board and Manitoba Health’s Health Information Privacy Committee. These committees do not require individual consent for research conducted using de-identified administrative data when reasonable safeguards to protect confidentiality and security of personal health information are in place.

## Results

**Fig. 1:**
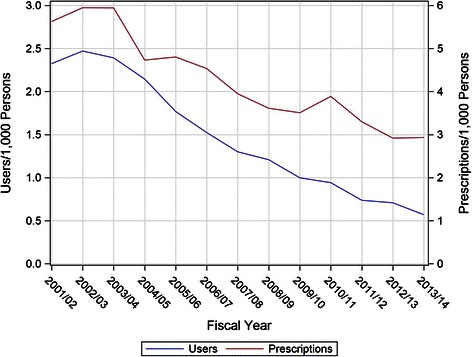
Meperidine utilization over time

We identified 9196 prescriptions for meperidine dispensed between April 1^st^, 2001 and March 31^st^, 2014 for 1990 persons aged 65 years and older in the DPIN database. In this overall population, there were 1.47 (95 % Confidence Interval (CI) 0.60–2.47) users per 1000 persons per year with 3.82 (95 % CI 3.27–4.38) prescriptions per 1000 persons per year. There was a marked decline in meperidine usage over the study period (Fig. 1).

The majority of meperidine users were female (1210; 60.8 %). When their first meperidine prescription was filled most users were 65 to 74 years (1160; 58.3 %) with a smaller number of users in the 75–84 (633; 31.8 %) and ≥85 (197; 9.9 %) age groups. The DDDs of meperidine dispensed declined with increasing age. The 65–74 age group had a mean rate of 50.2 (95 % CI 44.4–56.0), with 26.9 (95 % CI 22.2–31.5) for the 75–84 age group, and 17.8 (95 % CI 11.5–24.1) DDDs/1000-person-years in the 85 plus age group. Annual rates were found to be different between age groups (F _(2, 36)_ = 42.23; *p* < 0.0001), and pairwise comparisons showed significant differences between all age groups.

At the level of the user, the mean number of prescriptions per user per year was found to be 3.0 (95 % CI 2.8–3.2) prescriptions per year, representing 26.4 (95 % CI 24.0–28.8) DDDs per user per year. The mean overall daily dose of meperidine per prescription was 249.9 mg/day (95 % CI 242.3–257.4), and a mean prescription quantity of 8.80 DDDs/prescription (95 % CI 8.62–8.99).

When considering the prescribed dose of meperidine, 127 (6.4 %) of the users received at least one meperidine prescription above the maximum recommended dose of 600 mg per day. Overall, 336 (3.7 %) of the meperidine prescriptions exceeded the 600 mg maximum daily dose.

**Fig. 2:**
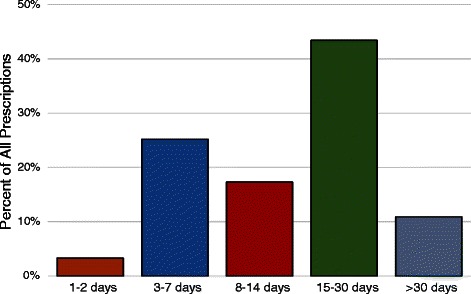
Distribution and duration of prescriptions

**Fig. 3:**
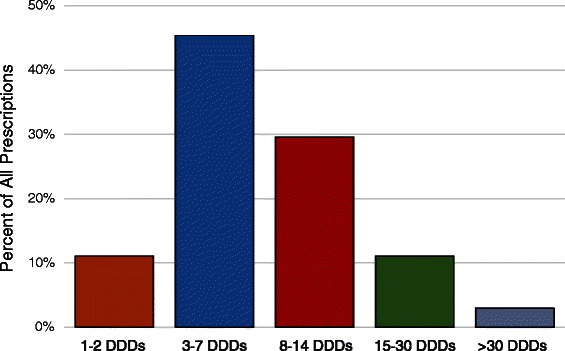
Distribution of DDDs per prescription

However, when considering the duration of treatment, prescription durations greatly exceeded the maximum 48 h recommended, with a mean length of 20.9 days (95 % CI 20.5–21.0). Overall, 96.7 % (8896) of prescriptions exceeded 2 days, and 54.3 % (4994) exceeded 2 weeks duration (Fig. 2). In terms of DDD standardized days of treatment, 88.9 % (8178) of all prescriptions dispensed were for quantities exceeding 2 DDDs, and 43.6 % (4005) of all prescriptions were for the equivalent of more than a week of therapy (≥8 DDDs) (Fig. 3).

**Fig. 4:**
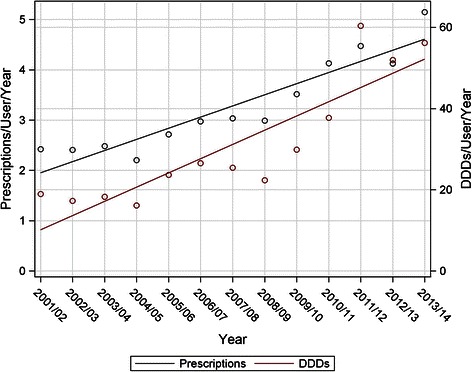
Meperidine prescriptions and DDDs per user over time

Between 2001 and 2013, the rate of meperidine use in this population declined steadily (Fig. 1). However, when the remaining users of meperidine were considered, the amount of meperidine per user was increasing (Fig. 4). Linear regression analysis of this data suggests a significant trend to increased intensity of use per individual user (Fig. 4). The mean number of prescriptions per user rose from 2.42 prescriptions/user/year in 2001/2002 to 5.15 prescriptions/user/year in 2013/2014 (F _(1, 11)_ = 73.81, *p* < 0.0001; *R*^*2*^ = 0.87). The amount of meperidine, expressed in DDDs/user/year, also rose steadily, from 18.98 in 2001/2002, to 56.14 DDDs/user/year in 2013/2014 (F _(1, 11)_ = 38.42, *p* = 0.0001; *R*^*2*^ = 0.78). Additionally, the mean number of DDDs per prescription increased from 7.84 DDD/prescription to 10.90 from 2001 to 2013 (peaking in 2011 at 13.47) (F _(1, 11)_ = 16.25, *p* = 0.002).

Less than 5 % (37/743) of the prescribers were responsible for over 50 % of all meperidine prescriptions (4612 out of 9196 prescriptions). This high prescribing group (the top 5 % of prescribers) wrote an average of 125 prescriptions per prescriber in contrast to the remaining prescribers (i.e. the remaining 95 % of meperidine prescribers) who averaged just 6.5 prescriptions per prescriber.

**Fig. 5:**
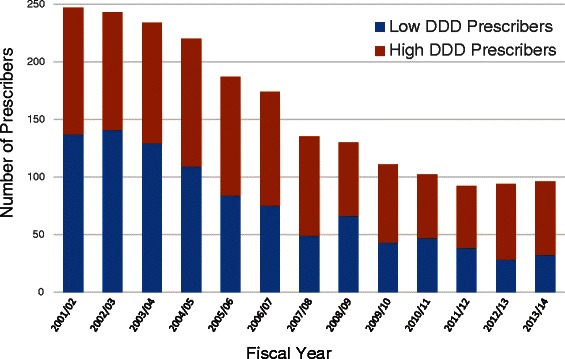
Meperidine prescribers

The number of prescribers of meperidine declined dramatically from 247 prescribers to 96 prescribers by the end of the study (Fig. 5). The number of low DDD prescribers declined more, from 137 to 32 prescribers, than high DDD prescribers group which fell from 110 to 64 prescribers. As a result, the proportion of high DDD prescribers has risen steadily across the study period from 44.5 % in 2001/02 to 66.7 % in 2013/14.

## Discussion

At a population level, there has been a marked decline in the use of meperidine by individuals 65 years and older (Fig. 1). This is reassuring given that a key message of the safety warnings was to avoid meperidine in older adults. Unfortunately the nature of the remaining small amount of use is concerning. While only a small percentage (3.65 %) of prescriptions exceeded the recommended dosing limit of 600 mg per day, the duration of prescriptions almost universally exceeded the recommended limit of 48 h (96.7 % of prescriptions). Overall the average DDDs per prescription (8.80) is the equivalent of just over seventy 50 mg tablets. The number of prescriptions and DDDs per user increased over the study period (Fig. 4), indicating an increasing level of presumably inappropriate use.

Many utilization studies have considered meperidine use in the hospital setting [7, 18–20] with a smaller number of studies considering the overall population use in the community [21, 22]. To the best of our knowledge, this is the first study to consider population level data exclusively in the higher risk older adult population. Fischer et al conducted a review of opioid analgesic use in Canada from 2005 to 2010 by extrapolating the data from a representative sample of retail pharmacy prescribing records [22]. They found that Canadian average use of meperidine fell from 60 to 43 DDD/1000 persons per year over their study period. Manitoba had among the lowest levels of use in Canada with use falling from 34 to 30 DDD /1000 population per year. These values correspond reasonably well to our previous assessment of meperidine use in the overall population with use falling from 46 to 31 DDD/1000 persons per year from 2001 to 2010 [23]. In this study, meperidine use in the population 65 years of age and older at 26.4 DDD/1000 persons per year was considerably lower than values previously reported for the overall population.

This low and declining level of population use has been previously shown in Nova Scotia, Canada [21]. That study found that only 30 (2.4 %) prescribers in the province were responsible for 40 % of the meperidine prescribed. A very similar concentration of meperidine prescribing was found in this study (5 % prescribers were responsible for 50 % of prescribing). Previous study in Manitoba has also shown that the number of prescribers of meperidine has been falling with less than 4 % of eligible prescribers prescribing meperidine to the broader population [23]. This is also evident in the absolute decline in the number of meperidine prescribers in Manitoba (Fig. 5). The higher decline in low DDD prescribers relative to high DDD prescribers may provide an explanation for the paradoxical rise in DDDs and prescriptions per user/year (Fig. 4). Prescribers who are sensitive to safety warnings may use lower quantities, and ultimately elect to discontinue use of meperidine in older adults. Prescribers who have ignored the safety warnings may tend to prescribe higher DDDs and continue to not only prescribe meperidine but to prescribe meperidine in large amounts. In an attempt to target this small resistant group, direct education and feedback to these prescribers has been shown to have some impact in reducing meperidine use [21].

There are some limitations to this study. The data available is limited to prescription filling records from community pharmacies. No information on meperidine use in hospital is available. However, given that many hospitals have removed meperidine from their formularies and taken efforts to restrict its use, hospital-based meperidine use is expected to be limited [19, 20]. The DPIN records provide only records of filling a prescription and do not assess if the medication was actually taken. This is a common limitation of utilization studies that make use of administrative data [24, 25]. The days supply field in the prescription records is manually entered by pharmacists and may not always be a reliable estimate of prescription duration. This limitation was addressed by also estimating the prescription duration based on the quantity of medication dispensed and its equivalent as DDDs. Safety warnings allow that meperidine use may be permissible for limited durations when patients have an allergy to all other available opioids. It is expected that this circumstance would be rare, but our records did not provide allergy information.

## Conclusions

Safety warnings and increased clinical awareness of the problems with meperidine in older adults have resulted in dramatic declines in the prescribing of meperidine to those who are 65 year of age or older. However, the small amount of remaining use appears to be less safe with an increase in the DDDs per user per year. This appears to be primarily related to a small number of high DDD prescribers that have not responded to past safety warnings. Educational initiatives directed at the small group of prescribers may be helpful [21]. Given that safer opioid alternatives exist for the vast majority of these patients, another approach may be to remove insurance coverage for meperidine for those over the age of 65 [26]. This may provide a further incentive to avoid meperidine in this sensitive population.

### Availability of data and materials

In accordance with the terms and conditions of the data providers, data files cannot be shared to ensure adequate protection of personal health information under Manitoba’s Personal Health Information Act.

## Abbreviations

ANOVA: Analysis of variance
CI: Confidence interval
DDD: defined daily dose
DPIN: Drug Program Information Network
ISMP: Institute for Safe Medication Practices
MCHP: Manitoba Centre for Health Policy
OR: odds ratio
